# Influence of Fresh Palm Fruit Sterilization in the Production of Carotenoid-Rich Virgin Palm Oil

**DOI:** 10.3390/foods10112838

**Published:** 2021-11-17

**Authors:** Nik Suhaimi Mat Hassan, Md. Sohrab Hossain, Venugopal Balakrishnan, Mark Harris Zuknik, Muliadi Mustaner, Azhar Mat Easa, Adel Al-Gheethi, Ahmad Naim Ahmad Yahaya

**Affiliations:** 1 School of Industrial Technology, Universiti Sains Malaysia (USM), Gelugor 11800, Malaysia; nik.suhaimi@simedarbyplantation.com (N.S.M.H.); zuknik@usm.my (M.H.Z.); azhar@usm.my (A.M.E.); 2 Sime Darby Plantation Research, R&D Centre—Carey Island, Lot 2664 Jalan Pulau Carey, Pulau Carey 42960, Malaysia; muliadi.mustaner@simedarbyplantation.com; 3 Institute for Research in Molecular Medicine (INFORMM), Universiti Sains Malaysia, Gelugor 11800, Malaysia; venugopal@usm.my; 4 Micro-Pollutant Research Centre (MPRC), Department of Civil Engineering, Faculty of Civil Engineering and Built & Environment, Universiti Tun Hussein Onn Malaysia, Batu Pahat 86400, Malaysia; adel@uthm.edu.my; 5 Department of Green Chemistry and Sustainable Development Engineering Technology, Malaysian Institute of Chemical and Bioengineering Technology, Universiti Kuala Lumpur, Kuala Lumpur 78000, Malaysia

**Keywords:** virgin palm oil, cold-press extraction, sterilization, oil palm fruits

## Abstract

Palm oil is known to be rich in carotenoids and other phytonutrients. However, the carotenoids and phytonutrients degrade due to high heat sterilization of oil palm fruits. The present study was conducted to produce carotenoid-rich virgin palm oil (VPO) using cold-press extraction. Herein, the influence of sterilization of oil palm fresh fruits in the production of cold-pressed VPO was determined with varying sterilization temperatures, times, and amounts of palm fruits in sterilization. The experimental sterilization conditions were optimized using response surface methodology (RSM) based on the maximum VPO yield and minimum FFAs in cold-pressed VPO. The optimal sterilization experimental conditions of oil palm fruits were determined to be a sterilization temperature of 62 °C, a time of 90 min, and an amount of oil palm fruits of 8 kg. Under these experimental conditions, the maximum cold-pressed VPO yield and the minimal content of free fatty acids (FFAs) obtained were 27.94 wt.% and 1.32 wt.%, respectively. Several analytic methods were employed to determine cold-pressed VPO quality and fatty acids compositions and compared with the crude palm oil. It was found that cold-pressed VPO contains higher carotenoids (708 mg/g) and unsaturated fatty acids compared with the carotenoid (343 mg/g) and fatty acid compositions in CPO. The findings of the present study reveal that the sterilization temperature potentially influences the carotenoid and nutrient contents in VPO; therefore, the optimization of the sterilization conditions is crucial to producing carotenoid- and phytonutrient-rich VPO.

## 1. Introduction

Palm oil is the most commonly consumed vegetable oil worldwide. The demand for palm oil production has increased immensely, and the global palm oil production was in surplus at 70 million metric tonnes since 2017 [1]. Generally, oil palm fruits consist of mesocarp fiber and palm kernel. The palm oil is extracted from the mesocarp fibers of the oil palm fruits. Conventional palm oil extraction requires multi-step processing, including oil palm fresh fruit bunch (OP-FFB) harvesting; OP-FFB sterilization; OP-FFB threshing; fruits digesting; oil extraction; oil clarification; and oil drying, storage, and dispatch [2,3]. In the oil palm fruit ripening process, the exocarp of the fruits becomes soft and easily attacked by lipolytic enzymes, increasing free fatty acids in the extracted oil due to hydrolysis, and the lipolytic enzymatic activity increase in detached fruits from the bunch [3,4]. Among the various palm oil processing steps, sterilization of OP-FFB is the most influential parameter in the production of palm oil [3].

In the palm oil mill, the FFA formation is inhibited with the aid of heat treatment known as the sterilization process. Steam-based sterilization is a commonly used heat treatment in the palm oil industry, operating at temperatures of 130–160 °C, sterilization times of 60–90 min, and elevated pressure [3]. The mechanical screw press method is the most common technique employed in palm oil industries for the extraction of oil from sterilized palm fruits. However, the extracted oil requires further refining processes, such as degumming, bleaching, and deodorization to remove wax, dirt, and other impurities [5]. The existing technologies employed in OP-FFB sterilization, palm oil extraction, and purification require high temperatures, resulting in degrading of the sensitive phytonutrients present in palm oil [3,6].

Cold press technology could be utilized as one of the effective alternative technologies to the conventional palm oil extraction technology that does not require temperature or any solvent to extract the oil [7,8,9]. Cold-pressed oil refers to the extraction of oil from fruits or seeds using a hydraulic or screw press [8]. The distinct advantages of the cold-press extraction technology are that this technology eliminates the oil refining processes and it does not deteriorate the nutrients and natural antioxidants present in the CPO [7,10]. Numerous studies have been conducted on the influence of cold-pressed extraction technology for the extraction of virgin coconut oil [10,11], chia seed oil [8], almonds oils [12], and rice bran oil [13]. Thus far, no study has yet implemented the cold-press extraction method to extract palm oil from oil palm fruits. However, African countries extracted palm oil from oil palm fruits by cooking the fruits to soften the mesocarp fiber, followed by mechanically pressing the softened fruits to extract the oil, similar to the cold press method [8].

Virgin palm oil (VPO) is a phytonutrient-rich palm oil that can be applied as food ingredients, pharmaceuticals, and cosmetics [6,14]. Generally, VPO is the improved version of palm oil and contains vitamin E, phytosterols, carotenoids, phenolic, medium-chain fatty acids, and water-soluble antioxidants [14]. Despite the wide application of palm oil in various products application, the production and uses of VPO in food, pharmaceutical, and cosmetics production are not well-known because of the lack of exposure to the functional properties of VPO, its usability, and health benefits. However, the production of VPO is challenging because the phytonutrients present in palm fruits are heat-sensitive, and therefore, the phytonutrients present in oil palm fruits deteriorate during the extraction of palm oil using conventional temperature-based palm oil processing technologies [6]. Thus, the implementation of low-temperature technologies in the sterilization of OP-FFB and in the extraction the oil from sterilized OP-FFB would be a practical implication to produce VPO.

Phan et al. [15] determined the influence of thermal cooking and ultrasonication pre-treatment on the extractability and quality of rice bran oil using the cold-press extraction method. The study reported that the oil extractability is potentially influenced by thermal cooking time, ultrasonic power, and duration. Moreover, the unsaturated fatty acids and oxidative stability were higher in the cold-press-extracted oil proceed with the short-period pre-treatment using thermal cooking. Abd Rashid et al. [6] produced VPO from oil palm mesocarp fiber using the low-heat aqueous enzyme extraction method. The study reported that temperature plays an effective role in the extraction of VPO and its phytonutrient content, color, and oxidative stability. The sterilization of oil palm fruits is the key factor in maintaining bioactive compounds and natural antioxidants in palm oil. Thus, the optimization of the temperature and duration of the sterilization process of oil palm fruits are crucial to obtaining phytonutrient- and carotenoid-rich VPO. Therefore, in the present study, the influence of the low-temperature sterilization process on the extraction of VPO from oil palm mesocarp fiber using the cold press method was determined. The sterilization process was optimized on the maximum oil yield and minimal free fatty acid (FFA) content in VPO using response surface methodology (RSM). Finally, various properties of VPO such as moisture, FFAs, peroxide values, phosphorus content, iodine number, carotenoids, saturated fatty acids, and unsaturated fatty acids content were determined and compared with the properties of crude palm oil (CPO) extracted using the conventional palm oil sterilization method.

## 2. Materials and Methods

### 2.1. Sample Collection and Preparation

The oil palm fresh fruits and crude palm oil (CPO) were collected from the Sime Darby Plantation, Carey Island, Selangor, Malaysia. The fresh palm fruits (bright red-orange color) were harvested from the young palm tree aged between 10–15 years. The average dimensions of the palm fruits were a diameter of 2.5 cm and a length of 4.0 cm. The collected fresh fruits were washed with tap water to remove trash and dirt.

### 2.2. Cold-Press Extraction Process

**Figure 1:**
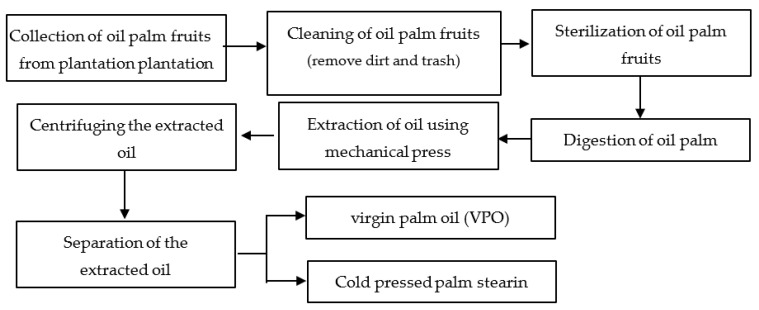
Process for the production of virgin palm oil using the cold-press extraction method.

Figure 1 shows the VPO extraction process using the cold-press extraction method. After cleaning, the oil palm fruits were sterilized with varying sterilizing time (30–120 min), temperature (30–60 °C), and amount of fruits (5–15 kg). The sterilization was conducted using a benchtop autoclave with a volume of 40 L and front loading (Astell Scientific Autoclave, Kent, UK). The sterilized palm fruits were digested at atmospheric pressure and a temperature of 60 °C for 15 min. Subsequently, oil was extracted from digested oil palm fruits using cold pressing at pressure 70 bar. The cold-pressed oil was then centrifuged at 1400 rpm and followed by separation using a two-phase decanter at 3000 rpm. After separation, the cold-pressed palm olein was collected for further analysis.

### 2.3. Design of Experiment for the Sterilization Oil Palm Fruits

The influence of oil palm fruit sterilization on the yield (wt.%) and FFA content was determined with varying sterilizing time (min), temperature (°C), and amount of fruits (kg). The central composite design (CCD) was utilized to design the sterilization experiments, and the sterilization process was optimized using RSM. In the sterilization process of oil palm fruits, the parameters of temperature, sterilization time, and amount fruits were independent variables, and the variables were coded following Equation (1).

(1) $$X=\frac{x-\left[x_{\max}+x_{\min}\right]/2}{\left[x_{\max}-x_{\min}\right]/2}$$

where *X* is the coded variable, *x* is the natural variable, *x*_max_ is the maximum level of the variables, and *x*_min_ is the minimum level of the variables. The maximum, intermediate, and minimum levels of the variables were coded as +1, 0, and −1, as shown in Table 1. Herein, +1.682 and −1.682 are the maximum and minimum axial points of the variables.

The sterilization process of VPO can be described by the second-order polynomial equation, as shown in Equation (2).

(2) $$Y=\beta_{o}+\sum_{i=1}^{n}\beta_{i}X_{i}+\sum_{i<j}^{n}\beta_{ij}X_{i}X_{j}+\sum_{i=1}^{n}\beta_{ii}X_{i}^{2}$$

where *Y* is the predicted yield of the VPO and FFA contents in VPO, *X* is the coded variable, *n* is the number of the coded variables. *β_o_* is the constant coefficient of intercept terms, *β_i_* is the constant coefficient of linear terms, *β_ij_* is the constant coefficient of interaction terms, and *β_ii_* is the constant coefficient of quadratic terms.

The experimental data of the sterilization process of VPO were analyzed and fit with the second order polynomial equation using Design expert software (ver.11, Stat-Ease Inc., Minneapolis, MN, USA). The accuracy of the regression model was evaluated by the coefficient of determination (*R*^2^) and adjusted coefficient of determination (*R*^2^*_adj_*). The three-dimensional graphical representation, also called response surface, was utilized to describe the interaction behavior on the maximum production of VPO and minimal FFA content in the extracted VPO using the cold-pressed method.

### 2.4. Analysis of VPO and CPO

Free fatty acid (FFA) in VPO was determined by the titration method according to AOCS Official Method Ca 5a-40 [16]. The yield of VPO was determined as the amount VPO (g) extracted from the sterilized fresh palm fruits, as shown in Equation (3).

(3) $$\mathrm{Yield}\left(\%\right)=\frac{\mathrm{Amount}\;\mathrm{of}\;\mathrm{VPO}\left(g\right)}{\mathrm{Amount}\;\mathrm{of}\;\mathrm{Fresh}\;\mathrm{palm}\;\mathrm{fruits}\;\left(g\right)}\times 100$$

The color of the VPO and CPO was determined in accordance with AOCS Official Method Cc 13 × 10^−92^ [17]. The oil was melted at 60 °C, and a Lovibond tintometer Model F (Wilts, England) was used to detect the color. The color of the oil was matched with a set of standard colored and numbered glasses ranging from 0 to 70 for red (R), from 0 to 70 for yellow (Y), and from 0 to 70 for blue (B). Peroxide Value (PV) in VPO and CPO was determined using the titration method according to AOCS Official Method Cd 8b-90 [18]. The oxidative stability of VPO and CPO was evaluated using the Rancimat Method. This is an accelerated aging method used to determine the oil and fat stabilities. The temperature was elevated at 110 °C, and rushing air was introduced during the analysis. The phosphorus content in CPO and VPO was determined using the colorimetric method following the Malaysian Palm Oil Board (MPOB) test method of 2005 [19], which involved ignition of the oil. The fatty acid compositions in CPO and VPO were determined using Gas chromatography (GC), and the analyses of the fatty acids were performed by converting the fatty acids to their respective methyl ester (FAME). The FAME was analyzed using GC equipped with a flame ionized detector (FID). About 0.1 μg of oil was injected into the capillary column (100 m × 0.25 mm, id., 0.25 μm particles; Supleco, Bellefonte, PA, USA) with a split ratio of 1:10. The initial oven temperature was set to 40 °C, heated up to 100 °C at a rate of 25 °C/min, and held for 25 min. Subsequently, the oven temperature further increased to 205 °C at a rate 10 °C/min and was held for 3 min, and eventually increased the temperature to 240 °C at a rate 10 °C/min and was held for 10 min. The temperature in the injector and detector was then maintained at 250 °C throughout the analyses. Helium was used as a carrier gas.

The cloud point of the VPO and CPO was determined following the MPOB test method [19]. The oil sample was filtered using a Whatman No.1 filter paper. Subsequently, the filtered oil was heated at 130 °C for 5 min. About 45 mL of heated oil was taken in a bottle and cooled in a water bath, which was thermostatically controlled below the expected could point. The oil was constantly stirred to avoid solidification of the fat catalyst on the bottom or side if the bottle. The cloud point was recorded as the temperature at which the immersed portion of the thermometer was no longer visible. The data were taken in triplicated, and average values was recorded. The Iodine values (IV) were determined following the MPOB test method [19] using Equation (4).

(4) $$IV=\frac{12.69M\left(V_{b}-V_{s}\right)}{W}$$

where *M* is the molarity of Na_2_SO_3_ solution, *V_b_* is the volume of Na_2_SO_3_ solution taken in the blank test, *V_S_* is the volume of Na_2_SO_3_ solution taken in the VPO test, and W is the weight (g) of the VPO taken for the analyses.

The carotenoid content in VPO and CPO was determined using the PORAM test method [20]. About 2 g of the oil was dissolved in 10 mL of n-hexane. The absorbance was measured at 450 nm using a UV–Vis spectrophotometer (CARY 60, Agilent). The calibration curves with the range of concentration 0.10–3.50 μg/mL were prepared for the working solution β-carotene. The calibration curves were plotted based on the least-squares method.

### 2.5. Statistical Analyses

All experiments were conducted in triplicate, and the results were presented as mean value ± standard deviation. The results on VPO yield, FFA content, and physicochemical properties of VPO and CPO were submitted to variance analyses (ANOVA). The mean values were analyzed with Tukey’s test analyses at 95% confidence level (*p* < 0.05) using MINIRAB software (ver. 16.1, Minitab^®^, Coventry, UK).

## 3. Results and Discussion

### 3.1. Effect of Sterilization Parameters in VPO Extraction

**Figure 2:**
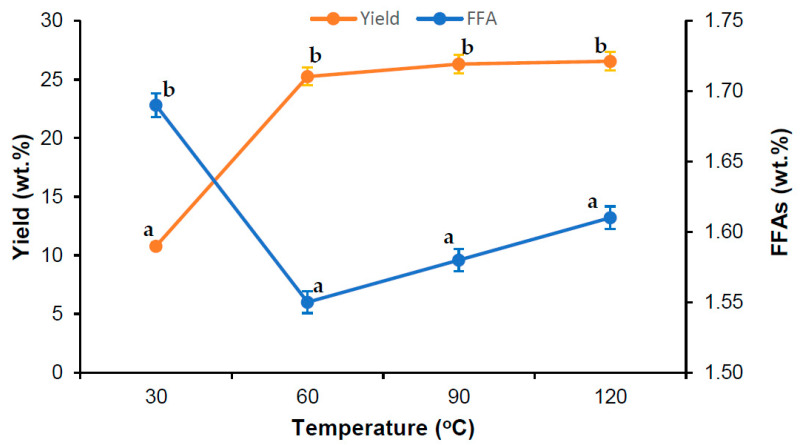
Effect of sterilization temperature in VPO extraction using cold press technology. Experimental conditions: time 60 min; amount of fresh palm fruits: 5 kg. Means of triplicates ± standard deviation. Means followed by different letters in the same line demonstrated significant difference by Tukey test (*p* < 0.05).

Sterilization is the pretreatment process for the extraction of oil from oil palm fruits. The sterilization aims to soften the fruits and degrades the lipolytic organisms that produce lipase enzymes in the extracted oil. The mesocarp fiber of oil palm fruits consists of cellulose, hemicellulose, and lignin [3]. Generally, hemicellulose (arabinan and xylan) surrounds the cell wall of the mesocarp fiber. During sterilization, the hemicellulose undergoes hydrolysis and breakdown of the sugars, resulting in soften the mesocarp fibers and releasing oil from the oil globule. The influence of sterilization temperature of the oil palm fruits on the oil yield (%) and FFAs (wt.%) in the cold-pressed VPO were determined as shown in Figure 2. It was found that percentage oil yield increased rapidly with increasing temperature from 30 °C to 60 °C; thereafter, the percentage oil increased slightly with a further increase of temperature from 60 °C to 120 °C. Conversely, the free fatty acid (FFA) content was sharply decreased with increasing temperature from 30 °C to 60 °C and thereafter increased gradually with a further increase in temperature from 30 °C to 120 °C. For a sterilization time of 60 min and amount of fresh palm fruits of 5 kg, the highest percentage of oil yield obtained was 26.55 wt.% at a temperature of 120 °C and the minimal FFA content was 1.55 wt.% in VPO obtained at 60 °C. However, the increase in VPO yield (%) and the reduction in the percentage of FFA content in VPO were statistically insignificant over the sterilization temperature of 60 °C. Therefore, the subsequent experiments on the sterilization of oil palm fruits for the VPO production could be conducted at a temperature of 60 °C.

**Figure 3:**
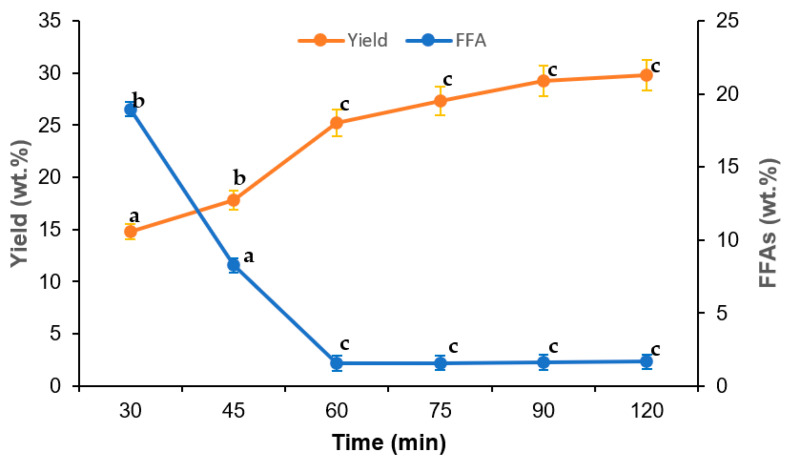
Effect of sterilization time in VPO extraction using cold press technology. Experimental conditions: temperature 60 °C; amount of fresh palm fruits: 5 kg. Means of triplicates ± standard deviation. Means followed by different letters in the same line demonstrated significant difference by Tukey test (*p* < 0.05).

**Figure 4:**
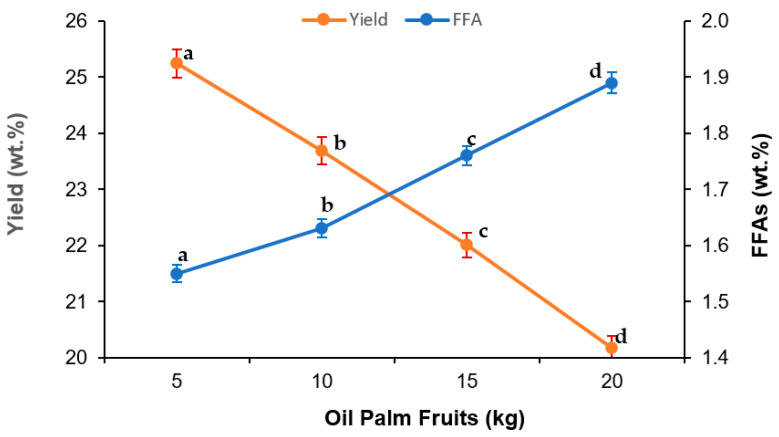
Effect of the amount of fresh fruit sterilization in VPO extraction using cold press technology. Experimental conditions: temperature 60 °C, time 60 min. Means of triplicates ± standard deviation. Means followed by different letters in the same line demonstrated significant difference by Tukey test (*p* < 0.05).

Figure 3 shows the influence of sterilization time on oil yield and FFA content in cold-pressed VPO. It was found that percentage oil yield sharply increased with increasing sterilization time from 30 min to 60 min; thereafter, the oil was increased gradually with further increases in sterilization time. The highest oil yield, about 30 wt.%, was obtained at 120 min sterilization, wherein it was about 25% at 60 min sterilization time. However, the VPO yield obtained at 90 min (29.22%) and 120 min (29.80%) were statistically insignificant. On the other hand, the percentage of FFA content in extracted VPO rapidly decreased with sterilization up to 60 min. The decrease in the FFA content in cold-pressed VPO was found negligible over the 60 min sterilization time. For a temperature of 60 °C and amount of palm fruits of 5 kg, the minimal FFA content was 1.55 wt.% in VPO obtained at 60 min sterilization time. The amount of oil palm fruits (kg) in sterilization also slightly influenced the oil yield and FFA content in VPO, as shown in Figure 4. It was found that the percentage VPO yield decreased with increasing amounts of oil palm fruit sterilization. Conversely, the FFA content in extracted VPO increased with an increasing amount of oil palm fruits. The decreases in oil yields and increased FFA content in cold-pressed VPO were attributed to the non-homogenous distribution of saturated steam heat with increasing amounts of oil palm fruits during sterilization. At higher amounts oil palm fruits in sterilization, some of the oil palm fruits might not be exposed to the saturated steam heat. Therefore, the mesocarp fiber might not soften enough for oil pressing, which reduces the VOP yield. Moreover, the lipolytic organisms in oil palm fruits might not be effectively inactive in the higher amount of oil palm fruits, which later increases the FFAs in the extracted oil [3].

Sterilization is a crucial parameter in palm oil extraction [21]. The quality of the palm oil is directly attributed to the sterilization efficiency of the palm fruits [21,22]. The most commonly utilized sterilization technology in palm oil extraction is the conventional steam-based sterilization technology, which operates at a temperature of 130–160 °C [4,22]. The purpose of utilizing such a high temperature in oil palm fruit sterilization is to detach the palm fruits from the bunch and to soften the mesocarp fibers. However, the extremely high temperature utilized in the oil palm fruit bunch sterilization degrades the phytonutrients present in the oil palm [4,6,22]. Moreover, there is rising concern about the FFA present in the extracted palm oil. As shown in Figure 2, the FFA content increased in VPO with increasing temperature over 60 °C of sterilization temperature. In the presence of active lipase, the triglyceride hydrolysis reaction rate increased at high temperatures, raising the FFA content in VPO and producing mono- and diglycerides [3,23]. Thus, its bears considerable interest to determine a low-temperature sterilization method to produce phytonutrient-rich palm oil.

### 3.2. Regression Model

Various factors potentially influence the oil palm fruits sterilization efficiency towards higher oil yield and low FFA content in the extracted oil. However, the conventional parameter at a time extraction technique does not accomplish the effect of the interaction of the parameters. Moreover, the response surface methodology (RSM) determines the interaction effects between the response variables and process factors. It is a less laborious and time-consuming technique to optimize process variables as it minimizes the number of experimental runs required to obtain adequate information for statistically acceptable results [24,25]. In the present study, RSM was utilized to determine the sterilization efficiency towards the maximum cold press VPO yield and the minimal FFA content in the extracted VPO. Moreover, the levels of variables (temperature, time, and amount of fruits) were chosen based on preliminary studies on the sterilization of oil palm fresh fruits, as shown in Figure 2, Figure 3 and Figure 4. CCD was utilized to design the experimental conditions of the variables, such as temperature, sterilization time, and amount of oil palm fruits. Table 2 shows the percentage of FFAs and VPO yield from the different experimental conditions of oil palm fruit sterilization.

The estimation of the regression coefficient of the oil palm fresh fruit sterilization on the cold-pressed VPO yield and FFA content is presented in Table 3. The regression coefficients of the intercept, interactions, and quadratic and linear terms were determined using the least squares method. The degree of significance was determined by evaluating the *p*-values for each variable at 95% confidence level (α = 0.05). It was found that the linear terms of temperature and amount of fruits; quadratic terms temperature * temperature, and amount of fruits * amount of fruits; interaction effects between temperature and amount of fruits had significant effects for the percentage FFA content in cold-pressed VPO. However, the linear terms of temperature and sterilization time, and the interaction effect between temperature and sterilization time had significant effects on the percentage VPO yield.

The predicted responses of the variables were determined with multiple regression analyses of experimental data. The test variables and response variables were related to second-order polynomial equations, as shown in Equations (5) and (6) for the yield and FFA content in cold-pressed VOP, respectively.

(5) $$Y_{Yield}=24.529+2.356X_{1}+1.409X_{2}-1.094X_{3}-0.37X_{1}^{2}-0.02X_{2}^{2}+0.119X_{3}^{2}-1.648X_{1}X_{2}-0.017X_{1}X_{3}+0.73X_{2}X_{3}$$

(6) $$Y_{FFAs}=1.639-3.5598X_{1}-0.0889X_{2}+0.7831X_{3}+2.9347X_{1}^{2}+0.0868X_{2}^{2}+0.4138X_{3}^{2}+0.085X_{1}X_{2}-0.794X_{1}X_{3}+0.68X_{2}X_{3}$$

where *Y* is the response of percentage VPO yield and FFA content in VPO.

The predicted values obtained from Equations (5) and (6) for the yield and FFA content in cold-pressed VOP, respectively, are shown in Table 2. Good agreement between the experimental values and predicted values for the percentage VPO yield and FFA content in VPO was found (Table 2). Table 4 shows the analysis of the variance (ANOVA) of the response surface quadratic model for the sterilization of the oil palm fruits on VPO yields and FFA content. The model was found significant for the percentage VPO extraction and FFA content in the cold-pressed VPO, indicating that the second-order polynomial equation was adequately fitted with the experimental data. Moreover, the insignificant lack of fit and minimal values of pure analyses for the percentage VPO yield and FFA content in the cold-pressed VPO reveals that the predicted model for the sterilization of oil palm fruits was adequate to obtain maximum VPO yield and minimal FFA content in extracted VPO. The goodness of fit of the regression equation was determined by assessing the regression coefficient (*R*^2^) and adjusted regression coefficient (*R*^2^*_adj_*) values. It was found that *R*^2^ and *R*^2^*_adj_* values for VPO yield (Equation (5)) and FFAs (Equation (6)) were 0.9761 and 0.9563, and 0.9824, and 0.9632, respectively. The *R*^2^ and *R*^2^*_adj_* values were close to 1, which indicates a high degree of correlation between the experimental and predicted values [24].

### 3.3. Response Surface Analyses

**Figure 5:**
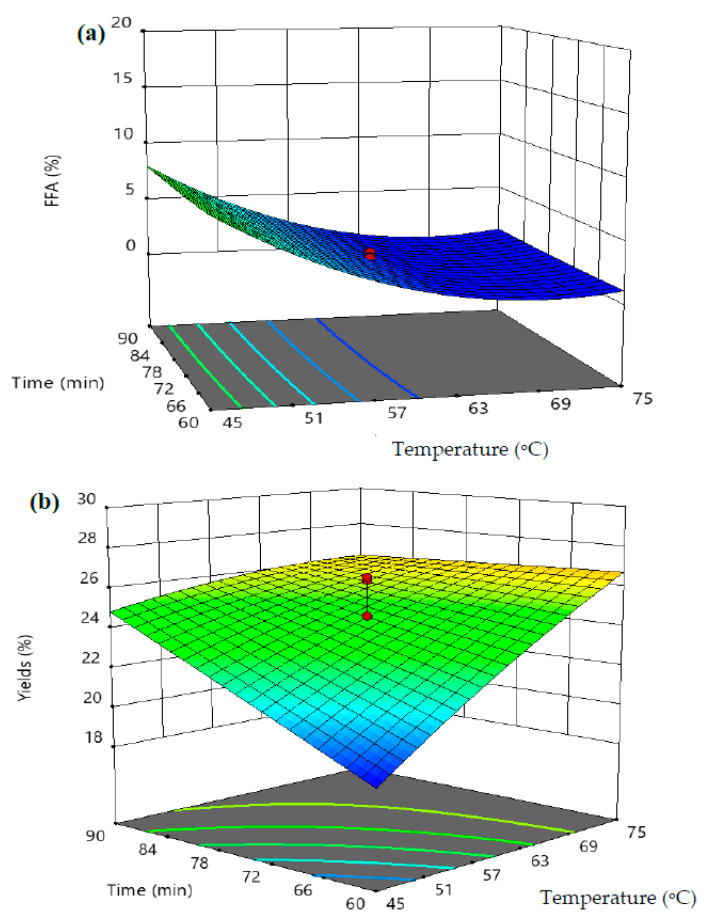
Interaction effects between the cold-pressed sterilization temperature and treatment time on FFAs in cold-pressed VPO (**a**) and VPO yield (**b**).

Figure 5 shows the interaction effects between temperature and sterilization time on forming the percentage FFAs (Figure 5a) and VPO yield (Figure 5b). It was found that the formation of FFAs decreased with increasing temperature from 45 °C to 65 °C; thereafter, the formation of the percentage of FFAs in extricated VPO was negligible with further increase in temperature over 65 °C. At a higher sterilization temperature, the increase in the treatment time did not influence the formation of FFAs in extracted VPO. Minimal percentage FFA formation was determined to be 1.51% at a temperature of 65 °C, the sterilization time of 60 min, and an amount of fresh palm fruits of 7.5 kg (Figure 5a). However, both sterilization temperature and time significantly influenced the percentage of VPO yield (Figure 5b). At a higher sterilization time, the increase in temperature from 45 °C to 75 °C increased the VPO yield. Similarly, the rise in the sterilization time increased the percentage of VPO yield. The maximum VPO yield, about 24% was obtained, at a temperature of 75 °C, a sterilization time of 90 min, and an amount of fresh palm fruits of 7.5 kg (Figure 5b).

**Figure 6:**
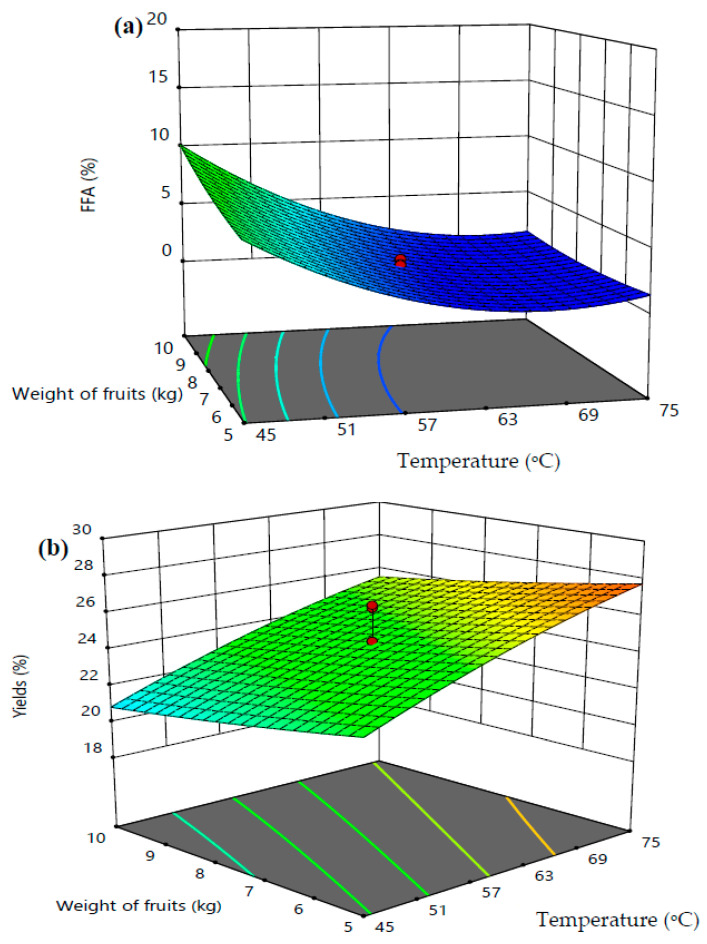
Interaction effects between the cold-pressed sterilization temperature and weight of palm fruits on FFAs in cold-pressed VPO (**a**) and VPO yield (**b**).

The interaction effects between the cold-pressed sterilization temperature and weight of palm fruits (kg) on the formation of FFAs in cold-pressed VPO and VPO yield are shown in Figure 6. It was observed that the increases in the weight of palm fruits (kg) in cold press sterilization at higher temperature and constant sterilization time (75 min) did not influence the percentage of FFA formation in extracted VPO (Figure 6a). Conversely, the percentage VPO yield increases with increasing temperature at a higher weight of fruits and constant sterilization time (Figure 6b). The highest VPO yield was about 28% at a temperature of 75 °C, weight of the fruits at 10 kg, and a sterilization time of 75 min.

**Figure 7:**
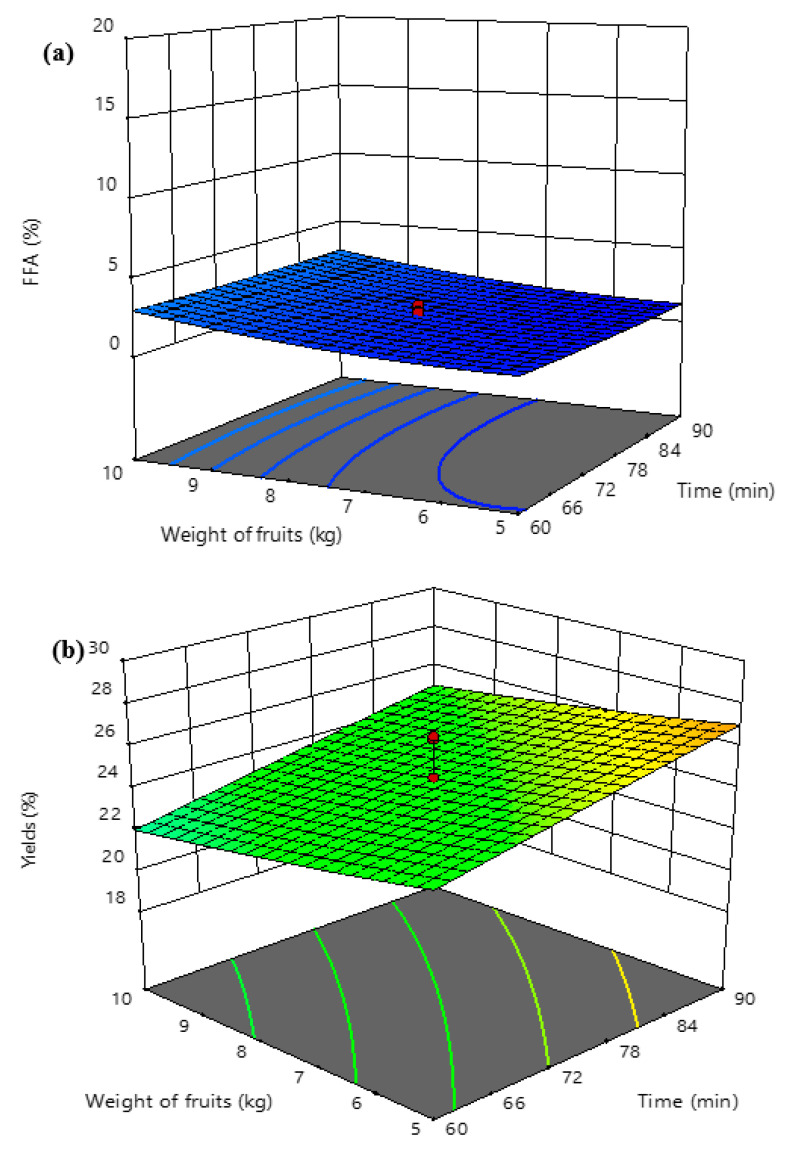
Interaction effects between the cold-pressed sterilization time and weight of fruits on FFAs in cold-pressed VPO (**a**) and VPO yield (**b**).

Figure 7 shows the interaction effects between the cold-pressed sterilization time and weight of the fruits on FFAs in cold-pressed VPO (Figure 7a) and VPO yield (Figure 7b). It was found that the increase in the weight of the fruits and sterilization time at constant temperature (60 °C) did not influence the formation of FFAs in the extracted VPO. Similarly, the increase in the weight of fruits at higher treatment times and the constant temperature had not influenced the percentage VPO yield (Figure 7b). However, the VPO yield was increased with increasing sterilization time from 60 min to 90 min at higher amounts of fruits and constant temperature. The optimal at about 27% VPO was obtained at a weight of fruits at 10 kg, a sterilization time of 90 min, and a temperature of 60 °C. Among the studied parameters, the sterilization temperature played an effective role in minimizing the formation of fatty acids in cold-pressed VPO and the extraction of VPO yield. Generally, oil palm fruits contain lipolytic microorganisms. Inefficient inactivation of these microorganisms allows for the production of lipase enzyme in the extracted oil, which subsequently acts as a catalyst to oxidize the oil and to produce FFAs [23]. The increase in temperature decreased the percentage FFA formation in cold-pressed VPO due to increasing inactivation of the lipolytic microorganisms. The inactivation of the microorganisms reached its maximum at 65 °C and obtained the minimal FFAs in cold-pressed VPO (Figure 7a). Moreover, the percentage of oil yield increased with increasing temperature because the higher temperature facilitates softening of the mesocarp fibers of the palm fruits, which is substantially easy in cold pressing oil, and hence increased the oil yield. However, the high temperature might degrade the heat-sensitive phytonutrients in the oil. Therefore, the optimization of sterilization conditions to obtain minimal FFAs and higher yield is crucial to obtain superior quality VPO.

The purpose of the oil palm fruit sterilization is to inactivate the enzymatic activity of the lipase enzyme and to soften the mesocarp fiber, resulting in easy cold pressing to extract VPO and to minimize the FFA content in extracted VPO. The using Design expert software (Ver.11, Stat-Ease Inc., Minneapolis, MN, USA) was utilized to optimize the experimental conditions of oil palm fresh fruit bunch sterilization for cold-pressed VPO production, as shown in Table 5. The optimized experimental conditions were determined to be a temperature of 62 °C, a treatment time of 90 min, and an amount of oil palm fruits at 8 kg. Under these optimal experimental conditions, the maximum VPO yield and FFA content in the cold-pressed VPO were determined to be 27.94 wt.% and 1.32 wt.%, respectively. Thus far, there are limited studies available in the literature on VPO extraction from oil palm fruits. Abd Rashid et al. [6] extracted VPO from oil palm fresh fruits using the low-heat aqueous-enzymatic extraction technique. It was found that the VPO yields from untreated and treated oil pam mesocarp fiber using pectinase enzyme at 40 °C for 24 h were 26.11% and 52.70%, respectively. However, the study had not determined the FFA content in the extracted VPO. The variation in the results might be due to the different extraction techniques utilized for the VPO extraction. However, the enzymatic extraction method is not cost-effective and is time-consuming, which hinder commercial-scale production.

### 3.4. Physicochemical Properties

The physicochemical properties, carotenoids, and fatty acid content in cold-pressed VPO from the oil palm fresh fruits sterilized using the optimized experimental condition were determined, as shown in Table 6. The VPO oil quality determined was compared with the crude palm oil (CPO), sterilized with conventional palm oil sterilization method, and extracted with mechanical screw pressed method. The carotenoid contents in VPO and CPO were 708 μg/g and 343 μg/g, respectively. The highest carotenoid content in cold-pressed VPO revealed that the optimized sterilization process did not affect the carotenoids content due to the relatively low-temperature sterilization method. The phosphorus and FFA content are the crucial properties used to determine any vegetable oil quality. It was found that the phosphorus content in the cold-pressed VPO was 1.92 mg/kg, which is almost similar to the phosphorus content of CPO. The FFA content in cold-pressed VPO (1.32 wt.%) was much lower than that in CPO (3.56 wt.%), revealing that the optimized low-temperature sterilization process potentially influences FFA formation in cold-pressed VPO. The color content in VPO was 30 Red/20 yellow. The minimal peroxide value of the cold-pressed VPO reveals that the VPO has higher oxidative stability. The higher IV values in cold-pressed VPO than in the CPO indicate that the optimized low-temperature sterilization method maintains the polyunsaturated fatty acid content in the cold-pressed VPO. The determination of the fatty acid compositions is vital in assessing vegetable oil’s nutritional value. Table 5 shows slight changes in the fatty acid compositions in VPO and CPO. Palmitic acid was the most predominant fatty acid in palm oil, followed by oleic acid and linoleic acid. Moreover, the saturated and unsaturated fatty acid content in CPO is almost 50:50. However, the saturated and unsaturated fatty acid content in VPO was determined to be 46:54. The cold-pressed VPO contains higher mono and polyunsaturated fatty acids than the CPO. Based on the VPO quality and fatty acids compositions analyses, it can be postulated that cold-pressed VPO is rich in carotenoids and other phytonutrients. Similarly, Abd Rashid et al. [6] reported that VPO extracted using low-temperature enzymatic extraction was rich in nutrients. Lietz et al. [26] reported that the VPO could be utilized as the richest dietary source because of its high carotenoid content.

## 4. Conclusions

The present study aimed to determine the influence of sterilization of oil palm fresh fruits in the production of cold-pressed VPO. It was found that the sterilization temperature and time potentially influenced the VPO yield and FFA content in cold-pressed VPO. The second-order quadratic model was adequately fitted with experimental data of the percentage VPO extraction and FFA content. The optimized experimental conditions were determined to be a temperature of 62 °C, a treatment time of 90 min, and oil palm fruits at 8 kg. Under these optimal experimental conditions, the maximum VPO yield and FFA content in the cold-pressed VPO were determined to be 27.94 wt.% and 1.32 wt.%, respectively. The determination of oil quality and fatty acid composition analyses revealed that the cold-pressed VPO contains higher carotenoids and unsaturated fatty acids. The carotenoid content in VPO and CPO were 708 μg/g and 343 μg/g, respectively. The findings of the present study reveal that the sterilization temperature potentially influenced the carotenoid and nutrient contents in VPO, and therefore, the optimization of the sterilization conditions is crucial to producing carotenoid- and phytonutrient-rich VPO.

## Figures and Tables

**Table 1:** The coded and uncoded levels of the independent variables.

Variable	Symbol	Factor Level				
		−1.682	−1	0	+1	+1.682
Temperature (°C)	*X*_1_	35	45	60	75	85
Sterilization time (min)	*X*_2_	50	60	75	90	100
Amount of fruits (kg)	*X*_3_	3.3	5	7.5	10	11.7

**Table 2:** Central composite design arrangement and responses.

Run	*X*_1_	*X*_2_	*X*_3_	FFAs (%)		Yield (%)	
				Actual	Predicted	Actual	Predicted
1	−1	−1	−1	7.25	7.30	18.81	19.99
2	1	−1	−1	1.58	1.60	27.33	28.04
3	−1	1	−1	6.74	6.81	26.32	25.96
4	1	1	−1	1.56	1.45	25.79	27.42
5	−1	−1	1	10.31	10.32	19.35	17.69
6	1	−1	1	1.62	1.44	25.34	25.67
7	−1	1	1	10.23	10.11	24.69	23.95
8	1	1	1	1.72	1.57	26.55	25.34
9	−1.682	0	0	15.98	15.93	18.6	19.52
10	1.682	0	0	3.75	3.95	28.32	27.45
11	0	−1.682	0	2.02	2.03	22.45	22.10
12	0	1.682	0	1.60	1.73	26.45	26.84
13	0	0	−1.682	1.56	1.49	28.6	26.71
14	0	0	1.682	3.91	4.13	21.09	23.03
15	0	0	0	1.58	1.64	26.35	24.53
16	0	0	0	1.65	1.64	24.56	24.53
17	0	0	0	2.18	2.32	21.65	24.53
18	0	0	0	1.76	1.64	26.51	24.53
19	0	0	0	1.68	1.64	24.55	24.53
20	0	0	0	1.01	1.64	23.56	24.53

**Table 3:** Regression coefficient and their significance of the quadratic model.

Term	Coefficient		Standard Error		*T*-Value		*p*-Value	
	FFAs	Yield	FFAs	Yield	FFAs	Yield	FFAs	Yield
Constant	1.639	24.529	0.123	0.766	13.29	32.04	0.000	0.000
*X*_1_	−3.559	2.356	0.082	0.508	−43.51	4.64	0.000	0.001
*X*_2_	−0.089	1.409	0.082	0.508	−1.09	2.77	0.303	0.020
*X*_3_	0.783	−1.094	0.082	0.508	9.57	−2.15	0.000	0.057
*X*_1_^2^	2.935	−0.370	0.079	0.494	36.85	−0.75	0.000	0.471
*X*_2_^2^	0.087	−0.020	0.079	0.494	1.09	−0.04	0.301	0.968
*X*_3_^2^	0.414	0.119	0.079	0.494	5.20	0.24	0.000	0.814
*X*_1_ *X*_2_	0.085	−1.648	0.107	0.664	0.79	−2.48	0.447	0.032
*X*_1_ *X*_3_	−0.794	−0.017	0.107	0.664	−7.43	−0.03	0.000	0.980
*X*_2_ *X*_3_	0.068	0.073	0.107	0.664	0.64	0.11	0.537	0.914

**Table 4:** Analysis of the variance (ANOVA) of the response surface quadratic model.

Source	Degree of Freedom	Sum of Squares		Mean Square		F-Value		*p*-Value	
		^a^ FFAs	^b^ Yield	FFAs	Yield	FFAs	Yield	FFAs	Yield
Model	9	311.71	143.44	34.63	15.94	379.32	4.53	0.0001	0.0136
Residual	10	0.9131	35.22	0.0913	3.52				
Lack of Fit	5	0.2049	18.75	0.0410	3.75	0.2894	1.14	0.9001	0.4452
Pure Error	5	0.7081	16.47	0.1416	3.29				
Total	19	312.62	178.66						

^a^*R*^2^ = 98.24%; *R*^2^*_adj_* = 96.32%. ^b^ *R*^2^ = 97.61%; *R*^2^*_adj_* = 95.63%.

**Table 5:** Optimized experimental conditions of oil palm fruit sterilization for maximum cold-pressed VPO production with minimal FFA content.

Parameters	Optimum Value	VPO Yield (wt.%)		FFAs (wt.%)	
		Predicted	Actual	Predicted	Actual
Temperature (°C)	62	28.61	27.94	1.27	1.32
Time (min)	90				
Weight of Fruits (kg)	8				

**Table 6:** Quality and fatty acid properties of virgin palm oil.

Properties	Unit	VPO	CPO
Color	Red/Yellow	30R/20Y	-
Moisture & Impurities	wt.%	0.12 ^a^ ± 0.01	0.10 ^a^ ± 0.01
Free fatty acids	wt.%	1.32 ^a^ ± 0.51	3.56 ^b^ ± 0.14
Peroxide value	meq/kg	0.42 ^a^ ± 0.08	3.39 ^b^ ± 0.11
Phosphorus	mg/kg	1.92 ^a^ ± 0.19	2.0 ^a^ ± 0.20
IV values	mg/kg	57.17 ^a^ ± 4.58	52.31 ^a^ ± 2
Cloud point	°C	9.61 ± 0.84	NA
Rancimat at 120 °C		5.63 ± 0.56	NA
Carotenoids	μg/g	708 ^a^ ± 24	343 ^b^ ± 12
**Fatty acids**			
Dodecanoic acid (C12:0)	%	0.04 ^a^ ± 0.01	0.341 ^b^ ± 0.01
Myristic (C14:0)	%	1.19 ^a^ ± 0.11	1.08 ^a^ ± 0.02
Palmitic (C16:0)	%	40.82 ^a^ ± 2.14	43.48 ^a^ ± 1.52
Palmitoleic (C16:1)	%	0.21 ^a^ ± 0.01	0.118 ^a^ ± 0.11
Stearic (C18:0)	%	3.84 ^a^ ± 0.11	4.436 ^b^ ± 0.16
Oleic (C18:1)	%	40.95 ^a^ ± 2.43	40.22 ^b^ ± 1.05
Linoleic (C18:2)	%	12.01 ^a^ ± 1.42	9.39 ^b^ ± 0.40
Linolenic (C18:3)	%	0.37 ^a^ ± 0.02	0.27 ^a^ ± 0.01
Arachidic (C20:0)	%	0.62 ^a^ ± 0.02	0.47 ^a^ ± 0.12
Saturated	%	46.51 ^a^ ± 1.21	49.82 ^b^ ± 0.56
Monounsaturated	%	41.16 ^a^ ± 1.65	40.34 ^a^ ± 1.32
Polyunsaturated	%	12.38 ^a^ ± 1.02	9.66 ^b^ ± 0.50

Means of triplicates ± standard deviation. Means followed by different letters in the same row demonstrated significant difference by Tukey test (*p* < 0.05).

## Data Availability

The datasets generated for this study are available on request to the corresponding author.
